# Modeling the effects of vector species composition and temperature on the risk of dengue virus

**DOI:** 10.1007/s11538-026-01650-2

**Published:** 2026-05-21

**Authors:** Suzanne L. Robertson, Rebeca de Jesús Crespo, Emma Beck, Lauren Beuerle, Patt Martin, Regan Stambaugh, Michael A. Robert

**Affiliations:** 1 Department of Mathematics and Applied Mathematics, Virginia Commonwealth University, Richmond, VA USA; 2 Department of Environmental Sciences, Louisiana State University, Baton Rouge, LA USA; 3 Department of Mathematics, University of Washington, Seattle, WA USA; 4 Department of Mathematics and Statistics, Elon University, Elon, NC USA; 5 Department of Mathematics, University of North Carolina Asheville, Asheville, NC USA; 6 Department of Mathematics and Statistics, Grinnell College, Grinnell, IA USA; 7 Department of Mathematics, Virginia Tech, Blacksburg, VA USA; 8 Center for the Mathematics of Biosystems (VT-CMB), Virginia Tech, Blacksburg, VA USA; 9 Center for Emerging, Zoonotic and Arthropod-borne Pathogens (CeZAP), Virginia Tech, Blacksburg, VA USA

**Keywords:** Stochastic epidemic model · Continuous-time Markov chain · Multitype branching process · Vector-borne disease · *Aedes aegypti* · *Aedes albopictus*

## Abstract

The risk of vector-borne disease is highly dependent on the local community composition of hosts and vectors, as well as the means by which disease is introduced into a susceptible population. The mosquito species *Aedes aegypti* and *Aedes albopictus* are both vectors of dengue virus, but differ in their biting preferences and ability to transmit the disease. The two species compete for habitat at the larval stage and their spatial distributions are highly heterogeneous, due in part to variability in factors such as temperature and resource quality affecting the outcome of competition and the resulting abundance of each species. In addition to affecting vector population dynamics, temperature also strongly affects multiple aspects of the disease transmission process. We present the basic reproduction number $R_0$ for a deterministic temperature-dependent transmission model between humans, *Ae. aegypti*, and *Ae. albopictus*, then develop a stochastic continuous-time Markov chain transmission model and determine the probability of disease extinction $\mathbb {P}_0$ for introduction by exposed or infectious humans, *Ae. aegypti*, or *Ae. albopictus*. We explore how both $R_0$ and $\mathbb {P}_0$ depend on a number of variables, including temperature, vector species composition, vector-host ratio, and mosquito biting behavior. We discuss our results in the context of changes in climate and neighborhood-level spread of mosquito populations and dengue.

## Introduction

For vector-borne diseases, such as dengue, risk of infection in humans depends strongly on the type and abundance of mosquito species present in an area, which in turn depends on environmental factors such as temperature. *Aedes aegypti* and *Aedes albopictus* are two vectors of dengue that share habitat requirements but differ in their biting behavior and ability to spread disease. These two mosquito species also differ in responses to temperature which impacts behavioral and life history traits differently for each species and plays a role in determining their distribution (Reinhold et al. 2018).

Distributions of both species overlap globally, and in these regions of overlap, *Ae. aegypti* and *Ae. albopictus* compete for resources and habitat, primarily artificial containers, at the larval stage (Kraemer et al. 2019; Laporta et al. 2023; Ding et al. 2018). There is significant heterogeneity in their spatial distributions; *Ae. aegypti* is typically associated with hotter, more urban areas while *Ae. albopictus* is associated with cooler, more vegetated areas (O’dell et al. 2025). *Ae. albopictus* has effectively displaced *Ae. aegypti* from many locations due to its generally superior competitive ability at the larval stage (O’Meara et al. 1995; Hawley et al. 1987; Lounibos 2007; Moore et al. 1999; Fader 2016). However, *Ae. aegypti* has a higher tolerance to heat and is able to survive at higher temperatures than *Ae. albopictus* (Reinhold et al. 2018). *Ae. aegypti* are often found in urban heat islands, areas of cities with little to no vegetation and high heat index (de Jesús Crespo R, Rogers RE 2021; Sangermano et al. 2025). Beyond survival, temperature also impacts many aspects of the mosquito life-cycle and disease transmission process – fecundity, larval development rates, length of the gonotrophic cycle, disease incubation rates, and transmission rates (Reinhold et al. 2018; Mordecai et al. 2017).

The presence of vegetation can lower temperatures, and also affect the basal resources available to mosquito larvae. Resource quality in turn impacts competition between *Ae. aegypti* and *Ae. albopictus* (Juliano 2010; Juliano et al. 2004; Yee et al. 2004; Camara et al. 2016; Lounibos and Juliano 2018). In particular, high quality resources alleviate the strong competitive effect of *Ae. albopictus* on *Ae. aegypti* and can promote coexistence of the two species (Beck et al. 2025). Within urban areas, heterogeneity in the type and amount of vegetation drives micro-environments differing in temperature and larval resources, which can result in heterogeneous spatial distributions of *Ae. aegypti* and *Ae. albopictus*, as was recently observed in New Orleans, Louisiana (de Jesús Crespo R, Rogers RE 2021). These micro-environments also exhibit significant variation in other factors influencing the vector-host ratio and vector-borne disease transmission, such as human population density and economic status.

While both *Ae. aegypti* and *Ae. albopictus* can transmit disease to humans, *Ae. albopictus* has been suggested to be a less competent vector of dengue, as well as other diseases such as Zika virus, due to lower transmission probabilities per bite (Whitehorn et al. 2015; Lozano-Fuentes et al. 2019; Vega-Rúa et al. 2014). *Ae. albopictus* is also a more opportunistically biting species than *Ae. aegypti* and may bite alternative hosts if available, resulting in a lower frequency of feeding on human hosts (Lambrechts et al. 2010; Sivan et al. 2015). In contrast, *Ae. aegypti* might take multiple bites per gonotrophic cycle, with each bite potentially spreading disease (Scott et al. 1993; Rezza 2012).

Beck et al. (2025) studied the effects of temperature and resource quality on the outcome of competition between *Ae. aegypti* and *Ae. albopictus*  and determined the resulting risk of infectious disease using a deterministic ordinary differential equation model for dengue transmission between humans and two vector species. Risk was measured by the basic reproduction number $R_0$, the expected number of secondary infections introduced into a susceptible population by a typical infected individual. They found that the outcome of competition (coexistence or competitive exclusion) and the resulting abundance of each species was dependent on both resource quality and the relative values of temperature-dependent fecundity and mortality rates. In turn, the value of $R_0$ depended on vector composition, temperature-dependent vector mortality rates, temperature-dependent disease-related parameters (host-vector and vector-host transmission probabilities, extrinsic incubation period length), and vector biting behavior. The baseline biting rate for each species assumed all bites were on humans, but alternative biting behaviors were also explored.

With a deterministic model, disease introduction either leads to an outbreak or the disease dies out depending on whether $R_0$ is above or below 1. For a stochastic model where variables are integer valued, an outbreak does not necessarily occur upon disease introduction even when $R_0 > 1$, and the probability that the disease dies out prior to resulting in a major outbreak depends on how the disease is introduced into the population. If the disease is introduced by an exposed individual, they may die prior to becoming infectious, and infectious individuals may recover or die prior to transmitting the disease. In regions presently naïve to dengue and related arboviruses, such as the southeastern United States, the primary means by which dengue is introduced into susceptible populations is exposed or infectious travelers returning from areas abroad where dengue is endemic. While *Ae. aegypti* and *Ae. albopictus* have limited flight ranges (Honório et al. 2003), it is also possible that disease could be introduced on a local neighborhood scale by exposed or infectious mosquitoes.

The probability of vector-borne disease extinction for different means of disease introduction was previously explored by Allen and van den Driessche (2013) and Maliyoni (2020) in models with a single species of host and vector, finding the probability of extinction for introduction by an exposed mosquito to exceed that of an infectious mosquito and the probability of extinction for introduction by an infectious host typically in between. Lloyd et al. (2007) developed methodology for determining disease invasion probabilities in a model with an arbitrary number of host and vector species, focusing on the case of 1 vector and 2 host species, where hosts are identical except for their abundance and vector biting rates. They found the highest probability of disease extinction to occur when vectors have no host preference and the disease is introduced by an infectious vector. Horton and Robertson (2024) also explored a model with transmission between 2 hosts and a single vector, finding the probability of an outbreak was typically greater when the disease was introduced by an infectious vector compared to an infectious host, since vectors were always assumed to find a host to bite while the likelihood of a single host being bitten was dependent upon both the vector-host ratio and vector biting preferences.

In this paper we extend the results of Beck et al. (2025) and explore how the local risk of dengue transmission depends on the composition of *Ae. aegypti* and *Ae. albopictus*, temperature, biting behavior, and how the disease is introduced into the population. Rather than explicitly modeling competition between vectors, we explore how dengue risk, as measured by the basic reproduction number and probability of disease extinction, changes with the overall vector abundance and the relative abundance of each species at different temperatures and for different assumptions about vector biting rates on humans. In the following sections, we present a deterministic transmission model that is a modification of Beck et al. (2025) with directly input vector abundance along with the basic reproduction number $R_0$, then derive a stochastic continuous-time Markov chain (CTMC) model and compute the theoretical probabilities of disease extinction resulting from disease introduction by each type of infected individual. We show how disease risk depends on total vector abundance, the relative abundance of *Ae. aegypti* versus *Ae. albopictus*, temperature, biting behavior, and the type of infected individual introducing the disease.

## Deterministic Model

Herein, we first describe the deterministic ordinary differential equations (ODE) model that underlies the stochastic modeling framework we describe in the next section. This mechanistic model is critical for the development of the continuous-time Markov chain model (CTMC) that we implement later in the study. A stage-structured deterministic model for vector-borne disease transmission incorporating competition between *Ae. aegypti* and *Ae. albopictus* at the juvenile stage and transmission between adult mosquitoes and humans was developed in Beck et al. (2025). The values of competition coefficients were assumed to depend upon the quality of available resources, while all other vector-related parameters were assumed to vary with temperature (fecundity, larval development rates, vector biting rates, extrinsic incubation periods, vector-host and host-vector transmission probabilities, and vector density-independent mortality rates). Human-related parameters were assumed to be independent of temperature.

Here we modify this model by removing the juvenile equations and eliminating explicit competition between mosquito species. The total adult population of each species is instead prescribed, denoted by $N_{v_1}$ for *Ae. aegypti* and $N_{v_2}$ for *Ae. albopictus*. Susceptible vectors of each species are found by subtracting the exposed and infectious populations from the total population size, which is assumed to remain constant. Dengue transmission can occur when a susceptible vector of either species bites an infectious human or when an infectious vector of either species bites a susceptible human. After a disease-transmitting bite occurs, the exposed individual must survive the incubation period of the virus in order to become infectious. Model equations are shown in (1), with temperature-dependent parameters shown as functions of *T*. We assume constant temperature throughout our analysis, so model (1) is autonomous. Parameters are defined in Table 1 and baseline parameter values are given in Appendix A.

1 $$\begin{aligned} \frac{ dE_{v_1}}{dt}= &  \left( \frac{b_1(T)\rho _{v_1}(T)I_h}{N_h}\right) S_{v_1}-\sigma _1(T)E_{v_1}-\mu _1(T)E_{v_1} \nonumber \\ \frac{dI_{v_1}}{dt}= &  \sigma _1(T)E_{v_1}-\mu _1(T)I_{v_1} \nonumber \\ \frac{ dE_{v_2}}{dt}= &  \left( \frac{b_2(T)\rho _{v_2}(T)I_h}{N_h}\right) S_{v_2}-\sigma _2(T)E_{v_2}-\mu _2(T)E_{v_2} \nonumber \\ \frac{dI_{v_2}}{dt}= &  \sigma _2(T)E_{v_{2}}-\mu _2(T)I_{v_2} \nonumber \\ \frac{dS_h}{dt}= &  \mu _h N_h-\left( \frac{b_1(T)\rho _{h_1}(T)I_{v1}+b_2(T)\rho _{h_2}(T)I_{v_2}}{N_h}\right) S_h-\mu _h S_h \nonumber \\ \frac{dE_h}{dt}= &  \left( \frac{b_1(T)\rho _{h_1}(T)I_{v_1}+b_2(T)\rho _{h_2}(T)I_{v_2}}{N_h}\right) S_h-\sigma _hE_h-\mu _h E_h \nonumber \\ \frac{dI_h}{dt}= &  \sigma _hE_h-r_hI_h-\mu _h I_h \nonumber \\ \frac{dR_h}{dt}= &  r_hI_h-\mu _h R_h \end{aligned}$$

and

$$\begin{aligned} S_{v_1}(t)= &  N_{v_1} - E_{v_1}(t) - I_{v_1}(t) \\ S_{v_2}(t)= &  N_{v_2} - E_{v_2}(t) - I_{v_2}(t) \end{aligned}$$

where $N_h = S_h(t) + I_h(t) + R_h(t)$ is constant. Model (1) has the disease free equilibrium $(S_{v_1}^*, E_{v_1}^*, I_{v_1}^*, S_{v_2}^*, E_{v_2}^*, I_{v_2}^*, S_h^*, E_h^*, I_h^*, R_h^*) = (N_{v_1}, 0, 0, N_{v_2}, 0, 0, N_h, 0, 0, 0)$.

**Table 1:** Parameter and variable descriptions for the disease transmission model (1). Species 1 is *Ae. aegypti* and species 2 is *Ae. albopictus*

$S_{v_i}$	Susceptible vectors of species *i*
$E_{v_i}$	Exposed vectors of species *i*
$I_{v_i}$	Infected vectors of species *i*
$N_{v_i}$	Total vectors of species *i*
$S_{h}$	Susceptible hosts
$E_h$	Exposed hosts
$I_h$	Infected hosts
$R_h$	Recovered hosts
$N_h$	Total host population
$b_i(T)$	Biting rate of species *i* on human hosts
$\rho _{v_i}(T)$	Probability of transmission from humans to species *i*
$\rho _{h_i}(T)$	Probability of transmission from species *i* to humans
$\sigma _i(T)$	Extrinsic incubation rate for species *i*
$\mu _i(T)$	Death rate of vector species *i*
$\sigma _{h}$	Intrinsic incubation rate
$r_h$	Human recovery rate
$\mu _h$	Birth/death rate of humans

The basic reproduction number, $R_0$, is the expected number of secondary cases arising from one typical infected individual introduced into a disease-free population. $R_0$ for model (1) is the same as that for the stage-structured model explicitly incorporating competition in Beck et al. (2025):

2 $$\begin{aligned} R_0=\sqrt{R_{V_1H}R_{HV_1}+R_{V_2H}R_{HV_2}} \end{aligned}$$

where

$$\begin{aligned} R_{V_1H}=\frac{b_1(T)\rho _{h_1}(T)\sigma _1(T)}{\mu _1(T)(\mu _1(T)+\sigma _1(T))}, \end{aligned}$$

$$\begin{aligned} R_{HV_1}=\frac{b_1(T)\rho _{v_1}(T)\sigma _hN_{v_1}}{N_h(\mu _h+r_h)(\mu _h+\sigma _h)}, \end{aligned}$$

$$\begin{aligned} R_{V_2H}=\frac{b_2(T)\rho _{h_2}(T)\sigma _2(T)}{\mu _2(T)(\mu _2(T)+\sigma _2(T))}, \end{aligned}$$

and

$$\begin{aligned} R_{HV_2}=\frac{b_2(T)\rho _{v_2}(T)\sigma _hN_{v_2}}{N_h(\mu _h+r_h)(\mu _h+\sigma _h)}. \end{aligned}$$

$R_{V_1H}$ is the expected number of new human infections resulting from an exposed *Ae. aegypti*, $R_{HV_1}$ is the expected number of new *Ae. aegypti* infections resulting from an exposed human, $R_{V_2H}$ is the expected number of new human infections resulting from an exposed *Ae. albopictus*, and $R_{HV_2}$ is the expected number of new *Ae. albopictus* infections resulting from an exposed human. We note that in the presence of only one species (e.g., $N_{v_1}=0$ or $N_{v_2}=0$), this expression for $R_0$ reduces to the $R_0$ obtained from a vector-host model with a single vector species.

## Stochastic Model

We next derive a continuous-time Markov chain (CTMC) model from the deterministic transmission model (1). We assume the model is time-homogeneous and satisfies the Markov property, so the future state of the model is only dependent on the current state (Allen 2010; Maliyoni 2020). The state variables are the same as those in model (1), but are now integer-valued random variables. A list of all possible state transitions is created from model (1), and the rates at which those transitions occur are given in Table 2.

**Table 2:** Transitions and Rates for CTMC Model. For each of ($S_{v_1}$, $E_{v_1}$, $I_{v_1}$, $S_{v_2}$, $E_{v_2}$, $I_{v_2}$, $S_h$, $E_h$, $I_h$, $R_h$), a 1 represents an increase by 1, -1 represents a decrease by 1, and 0 represents no change in the variable’s state from time *t* to time ($t + \Delta t$). Temperature dependent parameters are shown as functions of T. Parameter values are given in Appendix A and details are in Beck et al. (2025)

Event	Change in State	Rate
Exposure of *Ae. aegypti*	(-1,1,0,0,0,0,0,0,0,0)	$b_1(T)\rho _{v_1}(T)\frac{I_h}{N_h}S_{v_1}$
Infection of *Ae. aegypti*	(0,-1,1,0,0,0,0,0,0,0)	$\sigma _1(T)E_{v_1}$
Death of $E_{v_1}$	(0,-1,0,0,0,0,0,0,0,0)	$\mu _1(T) E_{v_1}$
Death of $I_{v_1}$	(0,0,-1,0,0,0,0,0,0,0)	$\mu _1(T) I_{v_1}$
Exposure of *Ae. albopictus*	(0,0,0,-1,1,0,0,0,0,0)	$b_2(T)\rho _{v_2}(T)\frac{I_h}{N_h}S_{v_2}$
Infection of *Ae. albopictus*	(0,0,0,0,-1,1,0,0,0,0)	$\sigma _2(T)E_{v_2}$
Death of $E_{v_2}$	(0,0,0,0,-1,0,0,0,0,0)	$\mu _2(T) E_{v_2}$
Death of $I_{v_2}$	(0,0,0,0,0,-1,0,0,0,0)	$\mu _2(T) I_{v_2}$
Recruitment of $S_h$	(0,0,0,0,0,0,1,0,0,0)	$\mu _h N_h$
Exposure of host	(0,0,0,0,0,0,-1,1,0,0)	$\left( \frac{b_1(T)\rho _{h_1}(T)I_{v_1}+b_2(T)\rho _{h_2}(T)I_{v_2}}{N_h}\right) S_h$
Infection of host	(0,0,0,0,0,0,0,-1,1,0)	$\sigma _h E_h$
Recovery of host	(0,0,0,0,0,0,0,0,-1,1)	$r_h I_h$
Death of $S_h$	(0,0,0,0,0,0,-1,0,0,0)	$\mu _h S_h$
Death of $E_h$	(0,0,0,0,0,0,0,-1,0,0)	$\mu _h E_h$
Death of $I_h$	(0,0,0,0,0,0,0,0,-1,0)	$\mu _h I_h$
Death of $R_h$	(0,0,0,0,0,0,0,0,0,-1)	$\mu _h R_h$

### Theoretical Probability of Disease Extinction

When susceptible populations are large enough, we can approximate the dynamics of the CTMC model near the disease-free equilibrium with a Galton-Watson multi-type branching process, a birth-death process for the number of infected individuals. Starting from a small number of infected individuals, the branching process has two possible outcomes, either quickly reaching the absorbing state of zero or growing exponentially. The former is called disease extinction (or a minor outbreak) and the latter is referred to as a major outbreak. Unlike $R_0$ for the deterministic model, the probability of disease extinction, denoted by $\mathbb {P}_0$, depends on which of the 6 types of infected individuals initially introduces the disease - exposed human (type 1), infectious human (type 2), exposed *Ae. aegypti* (type 3), infectious *Ae. aegypti* (type 4), exposed *Ae. albopictus* (type 5), or infectious *Ae. albopictus* (type 6).

Offspring probability generating functions (PGFs) for the distribution of new infections can be computed for each of these 6 infected types (Lahodny and Allen 2013). The offspring PGF for a single infected individual of type *i* ($i = 1,..., 6$) has the form:

3 $$\begin{aligned} f_i(\vec {u})=f_i(u_1,...,u_6) = \sum _{k_6=0}^{\infty }...\sum _{k_1=0}^{\infty }P_i(k_1,...,k_6)u_1^{k_1}...u_6^{k_6} \end{aligned}$$

where $\vec {u}=(u_1, u_2, u_3, u_4, u_5, u_6) \in [0,1]^6$ and $P_i(k_1,...,k_6)$ represents the probability that an infected individual of type *i* creates exactly $k_j$ new infections (i.e. offspring) of type *j*, $j = 1,..., 6$, when the susceptible population is at the disease free equilibrium (Allen 2017; Allen and Lahodny 2012).

The probabilities in the offspring PGFs are determined from the rates in Table 2 assuming the susceptible population is at the disease free equilibrium. An exposed human (type 1) will either survive the intrinsic incubation period of the disease to become infectious, or else die prior to becoming infectious. Survival results in the creation of one infectious human (type 2), so the probability of surviving the intrinsic incubation period, $\dfrac{\sigma _h}{\sigma _h+\mu _h}$, is multiplied by $u_2$ to the power one in the offspring PGF for type 1. Death prior to the end of the intrinsic incubation period results in no new infections, so the probability $\dfrac{\mu _h}{\sigma _h+\mu _h}$ is multiplied by all $u_j$ raised to the power zero, or 1. Thus the offspring PGF for exposed humans, assuming $E_h(0) = 1$ and $I_h(0) = E_{v_1}(0) = I_{v_1}(0) = E_{v_2}(0) = I_{v_2}(0) = 0$, is

4 $$\begin{aligned} f_1(\vec {u}) = \frac{\sigma _h u_2 + \mu _h}{\sigma _h + \mu _h}. \end{aligned}$$

An infectious human (type 2) can either transmit the disease to *Ae. aegypti* or *Ae. albopictus*, or else recover or die prior to infecting a mosquito. Transmission to *Ae. aegypti* results in the creation of two offspring - one of type 2, since the infectious human is still infectious after the transmission event occurs, and one of type 3, the newly exposed *Ae. aegypti*. Therefore the probability that an infectious human generates an *Ae. aegypti* infection, $\dfrac{\dfrac{b_1(T) \rho _{v_1}(T) N_{v_1}}{N_h}}{\dfrac{b_1(T) \rho _{v_1}(T) N_{v_1}}{N_h} + \dfrac{b_2(T) \rho _{v_2}(T) N_{v_2}}{N_h} + r_h + \mu _h}$, is multiplied by $u_2$ to the power one and $u_3$ to the power one in the offspring PGF. Similarly, transmission to *Ae. albopictus* results in two infected offspring, one of type 2 and one of type 5, and recovering or dying prior to transmitting the disease results in zero infectious offspring. The offspring PGF for infectious humans, assuming $I_h(0) = 1$ and $E_h(0) = E_{v_1}(0) = I_{v_1}(0) = E_{v_2}(0) = I_{v_2}(0) = 0$, is then

5 $$\begin{aligned} f_2(\vec {u}) = \dfrac{\dfrac{b_1(T) \rho _{v_1}(T) N_{v_1}}{N_h} u_2 u_3 + \dfrac{b_2(T) \rho _{v_2}(T) N_{v_2}}{N_h} u_2 u_5 + r_h + \mu _h}{\dfrac{b_1(T) \rho _{v_1}(T) N_{v_1}}{N_h} + \dfrac{b_2(T) \rho _{v_2}(T) N_{v_2}}{N_h} + r_h + \mu _h}. \end{aligned}$$

An exposed *Ae. aegypti* (type 3) can either survive the extrinsic incubation period, producing an infectious *Ae. aegypti* (type 4), or die prior to becoming infectious, resulting in zero infectious offspring. The offspring PGF for exposed *Ae. aegypti*, assuming $E_{v_1}(0) = 1$ and $E_h(0) = I_h(0) = I_{v_1}(0) = E_{v_2}(0) = I_{v_2}(0) = 0$, is then

6 $$\begin{aligned} f_3(\vec {u}) = \frac{\sigma _1(T) u_4 + \mu _1(T)}{\sigma _1(T) + \mu _1(T)}. \end{aligned}$$

An infectious *Ae. aegypti* (type 4) can either transmit the disease to a susceptible human, producing two offspring (one of type 4 and one of type 1) or die before transmitting the disease, producing zero infectious offspring. The offspring PGF for infectious *Ae. aegypti*, assuming $I_{v_1}(0) = 1$ and $E_h(0) = I_h(0) = E_{v_1}(0) = E_{v_2}(0) = I_{v_2}(0) = 0$, is then

7 $$\begin{aligned} f_4(\vec {u}) = \frac{b_1(T) \rho _{h_1}(T) u_1 u_4 + \mu _1}{b_1(T) \rho _{h_1}(T) + \mu _1(T)}. \end{aligned}$$

An exposed *Ae. albopictus* (type 5) can either survive the extrinsic incubation period, producing an infectious *Ae. albopictus* (type 6), or die prior to becoming infectious, resulting in zero infectious offspring. The offspring PGF for exposed *Ae. aegypti*, assuming $E_{v_2}(0) = 1$ and $E_h(0) = I_h(0) = E_{v_1}(0) = I_{v_1}(0) = I_{v_2}(0) = 0$, is then

8 $$\begin{aligned} f_5(\vec {u}) = \frac{\sigma _2(T) u_6 + \mu _2(T)}{\sigma _2(T) + \mu _2(T)}. \end{aligned}$$

An infectious *Ae. albopictus* (type 6) can transmit the disease to a susceptible human, thereby producing two offspring (one of type 6 and one of type 1) or die before transmitting the disease, producing zero infectious offspring. The offspring PGF for infectious *Ae. albopictus*, assuming $I_{v_2}(0) = 1$ and $E_h(0) = I_h(0) = E_{v_1}(0) = I_{v_1}(0) = E_{v_2}(0) = 0$, is then

9 $$\begin{aligned} f_6(\vec {u}) = \frac{b_2(T) \rho _{h_2}(T) u_1 u_6 + \mu _2(T)}{b_2 \rho _{h_2}(T) + \mu _2(T)}. \end{aligned}$$

A $6 \times 6$ matrix $\mathbb {M}$ can be constructed from the offspring PGFs where the (*j*, *i*) entry of $\mathbb {M}$ is the expected number of infections of type *j* produced by an infected individual of type *i* (Allen and Lahodny 2012) ($\mathbb {M}$ is shown in Appendix B). The system (4)-(9) always has the fixed point $\vec {u}=(1, 1, 1, 1, 1, 1)$ since $f_i(1,1,1,1,1,1) = 1$, $i = 1,..., 6$. If the spectral radius of $\mathbb {M}$ is less than 1, the branching process is subcritical and this is the only fixed point. In this case the probability of disease extinction is 1. If the spectral radius of $\mathbb {M}$ is greater than 1, the branching process is supercritical and the system has an additional fixed point $\vec {q}=(q_1,q_2,q_3,q_4,q_5,q_6)$, so $f_i(\vec {q}) = q_i$, with $0< q_j < 1$, $j = 1,...,6$ (Allen and Lahodny 2012). The probability of disease extinction is then $\mathbb {P}_0 = q_1^{n_1}q_2^{n_2}q_3^{n_3}q_4^{n_4}q_5^{n_5}q_6^{n_6}$ where $n_i$ is the initial number of infected individuals of type *i* (Allen and Lahodny 2012). When considering introduction by only a single type *i* individual, the probability of disease extinction reduces to $\mathbb {P}_0 = q_i$. We show in Appendix B that the Threshold Theorem of Allen and van den Driessche (2013) holds, and $R_0 < 1 (=1, >1)$ if and only if $\rho (\mathbb {M}) < 1 (=1, >1)$.

In the southern United States, dengue is likely to enter naive areas by travelers returning by plane from areas where the disease is endemic. Therefore type 1 or 2 individuals (exposed or infectious humans) are most likely to introduce the disease. Mosquitoes typically do not travel long distances, but since we are interested in determining risk at the neighborhood scale, it is possible a mosquito can travel between neighborhoods and introduce the disease to a new neighborhood. Therefore we consider results for disease introduction by both humans and mosquitoes.

## Results

We explore how the basic reproduction number $R_0$ for the deterministic model and the theoretical probability of disease extinction $\mathbb {P}_0$ for the stochastic CTMC model depend on vector community composition (overall vector abundance as well as relative abundance of each species), temperature, and biting behavior. The equation for $R_0$ is given in (2), and the fixed points of equations (4)-(9) are found numerically in Matlab and used to compute $\mathbb {P}_0$ as described in Section 3.1. For every parameter set, the deterministic model yields a single estimate of risk, $R_0$, while the stochastic model gives an estimate of risk, $\mathbb {P}_0$, following introduction by each of the 6 types of infected individuals.

We note the probability of extinction for introduction by an exposed vector of either species will always be higher than the probability of extinction for introduction by an infectious vector of the same species, since an exposed vector may die prior to becoming infectious. However, the difference between the probabilities of extinction for introduction by exposed and infectious vectors depends on the length of the extrinsic incubation period of the disease and the mortality rate, which both vary with temperature. Since humans have a much longer lifespan than mosquitoes, the length of the intrinsic incubation period of the disease is short in comparison and the chance of exposed humans dying before becoming infectious is negligible. Therefore probabilities of extinction for exposed and infectious humans are essentially identical and we only show $\mathbb {P}_0$ for infectious humans in the results to follow.

In the remainder of this section, we explore the effects of *Aedes* species composition, vector-host ratio, temperature, and biting behavior on disease risk. We first investigate the impacts of these variables independently while considering influences of temperature and biting rate assumptions before looking more closely at how combinations of these variables work together to impact disease risk. As in Beck et al. (2025), we consider either a single bite per gonotrophic cycle on humans for each species, referred to as unscaled biting rates, or 50% of bites on humans for *Ae. albopictus* and 2 bites per gonotrophic cycle on humans for *Ae. aegypti*, referred to as scaled biting rates.

### Vector species composition

**Fig. 1:**
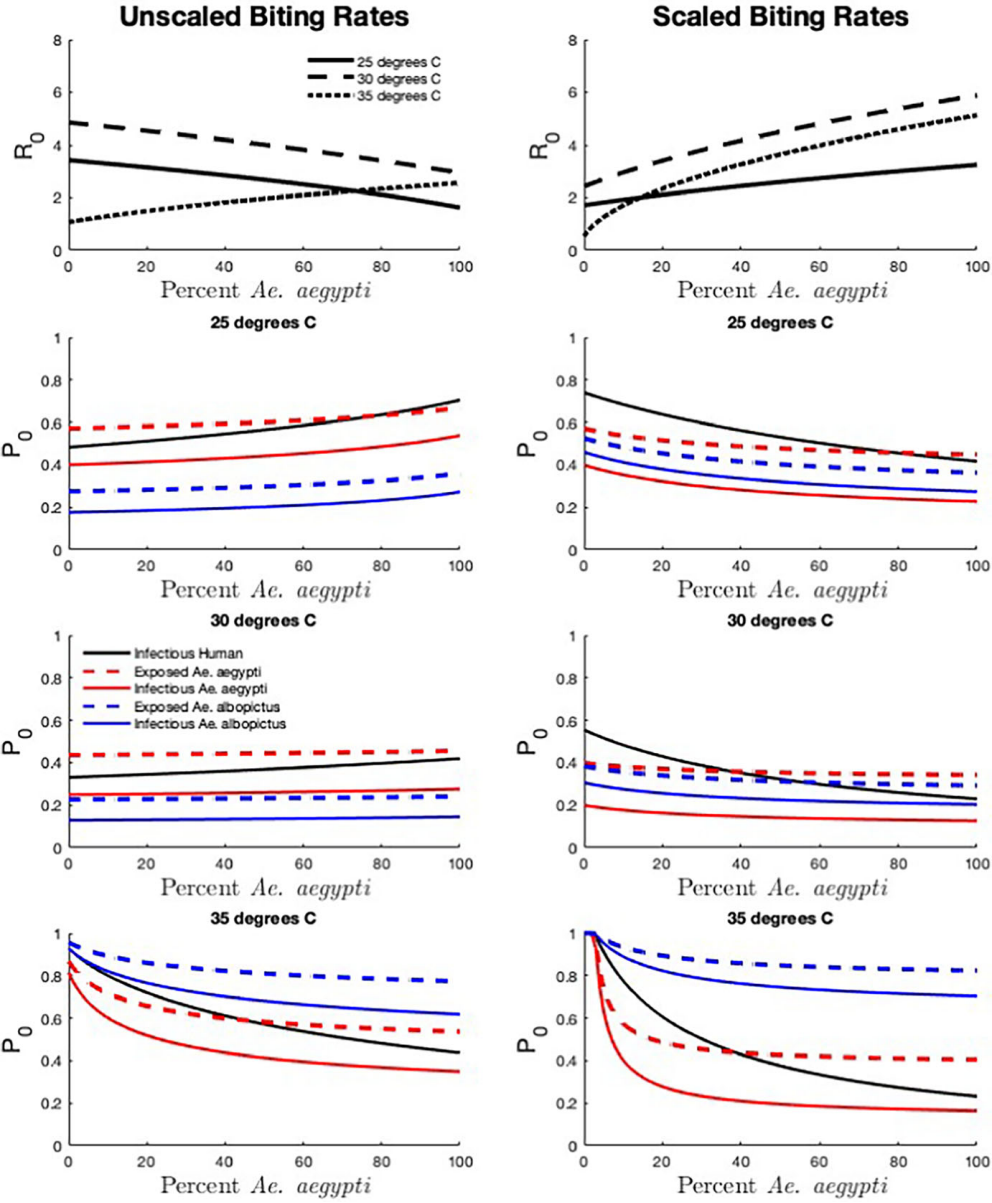
The effect of *Aedes* species composition on disease risk. Total vector abundance is held constant ($N_{v_1} + N_{v_2} = 200$) and the percentage of each species in the population is varied between 0 and 100. The basic reproduction number $R_0$ and the probability of disease extinction $\mathbb {P}_0$ (for introduction by infectious humans, exposed and infected *Ae. aegypti*, exposed and infected *Ae. albopictus*) are shown for three different temperatures (25, 30, and $35^{\circ }$C) and two biting rate scenarios (unscaled and scaled). $N_h=100$ for an overall vector-host ratio of 2. All additional parameter values are described in Appendix A (Color figure online)

To explore the effect of vector species composition on disease risk, we hold the total vector abundance, $N_{v_1} + N_{v_2}$, constant at 200 and vary the percentage of the population comprised of *Ae. aegypti* mosquitoes, $\dfrac{N_{v_1}}{N_{v_1} + N_{v_2}}$. We set $N_h = 100$ to maintain an overall vector-host ratio of 2, which is similar to ratios included in previous modeling studies (Manore et al. 2014; Robert et al. 2019). Results are shown in Figure 1 for three different temperatures – 25, 30, and $35^{\circ }$C – as well as two different biting rate scenarios.

We find that as the percentage of *Ae. aegypti* mosquitoes in the population increases, the effect on $R_0$ depends on both temperature and vector biting behavior. For unscaled biting rates, $R_0$ decreases with the percentage of *Ae. aegypti* in the population for lower temperatures, but increases at higher temperatures. When biting rates are scaled, $R_0$ increases with increased *Ae. aegypti* in the population regardless of temperature. For unscaled biting rates, temperature affects the direction of relationship between $\mathbb {P}_0$ and the percentage of *Ae. aegypti* in the population. As the percent of *Ae. aegypti* in the population increases, $\mathbb {P}_0$ increases for all types of introductions we considered at $25^{\circ }$C but decreases at $35^{\circ }$C. At $30^{\circ }$C, increasing the percent *Ae. aegypti* in the population has little impact on $\mathbb {P}_0$ associated with introductions by mosquitoes despite leading to substantial decreases in $R_0$, and only leads to a subtle increase in $\mathbb {P}_0$ following introductions by infectious humans. For scaled biting rates, the temperatures we consider here did not impact the directional relationship between the percent *Ae. aegypti* and $\mathbb {P}_0$. Notably, temperature has a strong influence on the relative values of $\mathbb {P}_0$ for introduction by each type of infected individual as the percent of *Ae. aegypti* in the population increases.

Overall, the probability of extinction for introduction by humans varies more with percent *Ae. aegypti* than for introduction by any other type of infectious individual, especially at higher temperatures. At $35^{\circ }$C, $\mathbb {P}_0$ for introduction by humans varies from 0.9 (100% *Ae. albopictus*) to 0.5 (100% *Ae. aegypti*) for unscaled biting rates and 1 (100% *Ae. albopictus*) to 0.25 (100% *Ae. aegypti*) for scaled biting rates. For the 2:1 vector-host ratio we consider, the probability of extinction is always lowest for introduction by a vector — *Ae. albopictus* for unscaled biting rates at $25^{\circ }$C and $30^{\circ }$C, and *Ae. aegypti* for unscaled biting rates at $30^{\circ }$C and scaled biting rates at 25, 30, and $35^{\circ }$C.

### Total Vector Abundance

**Fig. 2:**
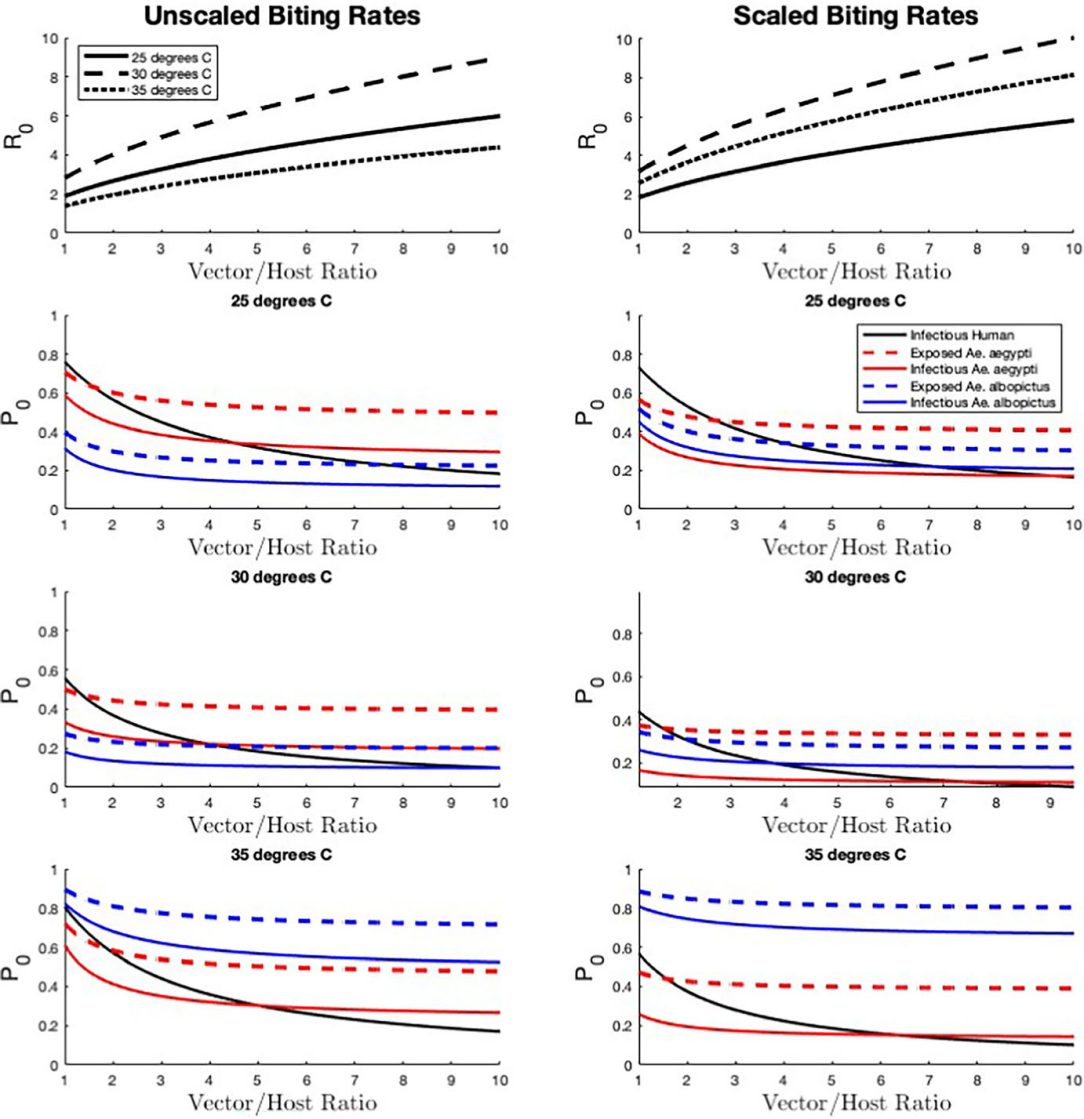
The effect of vector-host ratio on disease risk. Here $N_h = 100$ and we increase the overall vector abundance $N_{v_1}+N_{v_2}$ from 100 to 1000 (so the vector/host ratio varies from 1 to 10) while maintaining an equal abundance of each vector species (50% *Ae. aegypti* and 50% *Ae. albopictus*). The basic reproduction number $R_0$ and the probability of disease extinction $\mathbb {P}_0$ (for introduction by infectious humans, exposed and infected *Ae. aegypti*, exposed and infected *Ae. albopictus*) are shown for three different temperatures (25, 30, and $35^{\circ }$C) and two biting rate scenarios (unscaled and scaled). All additional parameter values are described in Appendix A (Color figure online)

Next we explore the effect of increased overall vector abundance, $N_{v_1}+N_{v_2}$, and thus the vector-host ratio, on $R_0$ and $\mathbb {P}_0$. We again consider three temperatures (25, 30, and $35^{\circ }$C) and both scaled and unscaled biting rates, and we assume the population is always composed of 50% *Ae. aegypti* and 50% *Ae. albopictus*. $R_0$ increases with vector-host ratio for all temperatures for both biting rate scenarios (Figure 2). For both scaled and unscaled biting rates, $R_0$ is greatest at $30^{\circ }$C, but for unscaled biting rates $R_0$ is lowest at $35^{\circ }$C while for scaled biting rates $R_0$ is lowest at $25^{\circ }$C.

**Fig. 3:**
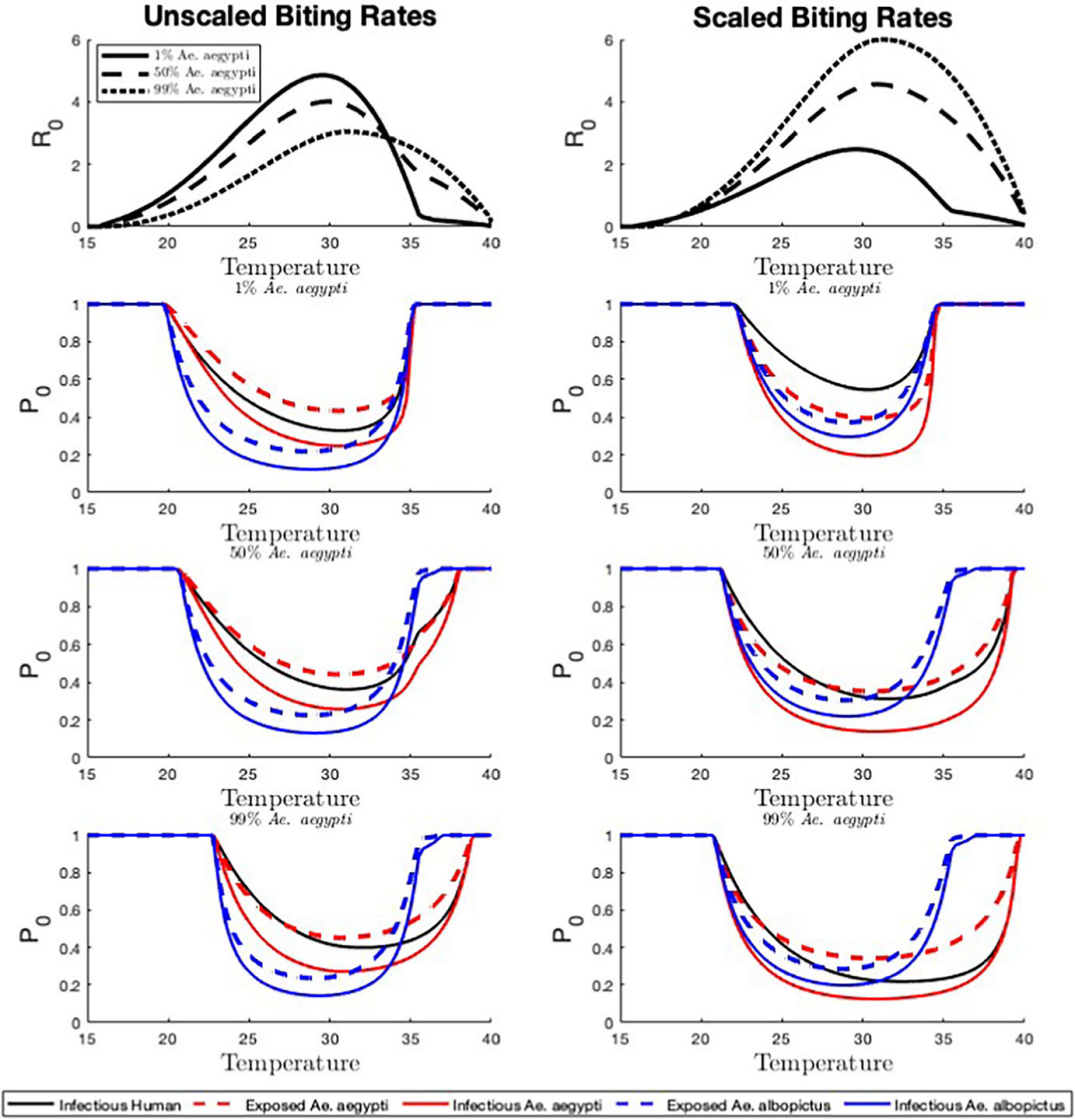
The effect of temperature on disease risk. We set $N_{v_1} + N_{v_2} = 200$ and $N_h=100$ for a baseline vector-host ratio of 2 with either a 1:99, 50:50, or 99:1 ratio of *Ae. aegypti* to *Ae. albopictus* and vary temperature from 15 to $40^{\circ }$C. $R_0$ and the probability of disease extinction $\mathbb {P}_0$ (for introduction by infectious humans, exposed and infected *Ae. aegypti*, exposed and infected *Ae. albopictus*) are shown for three different vector compositions (1%, 50%, and 99% *Ae. aegypti*) and two biting rate scenarios (scaled and unscaled). All additional parameter values are described in Appendix A (Color figure online)

**Fig. 4:**
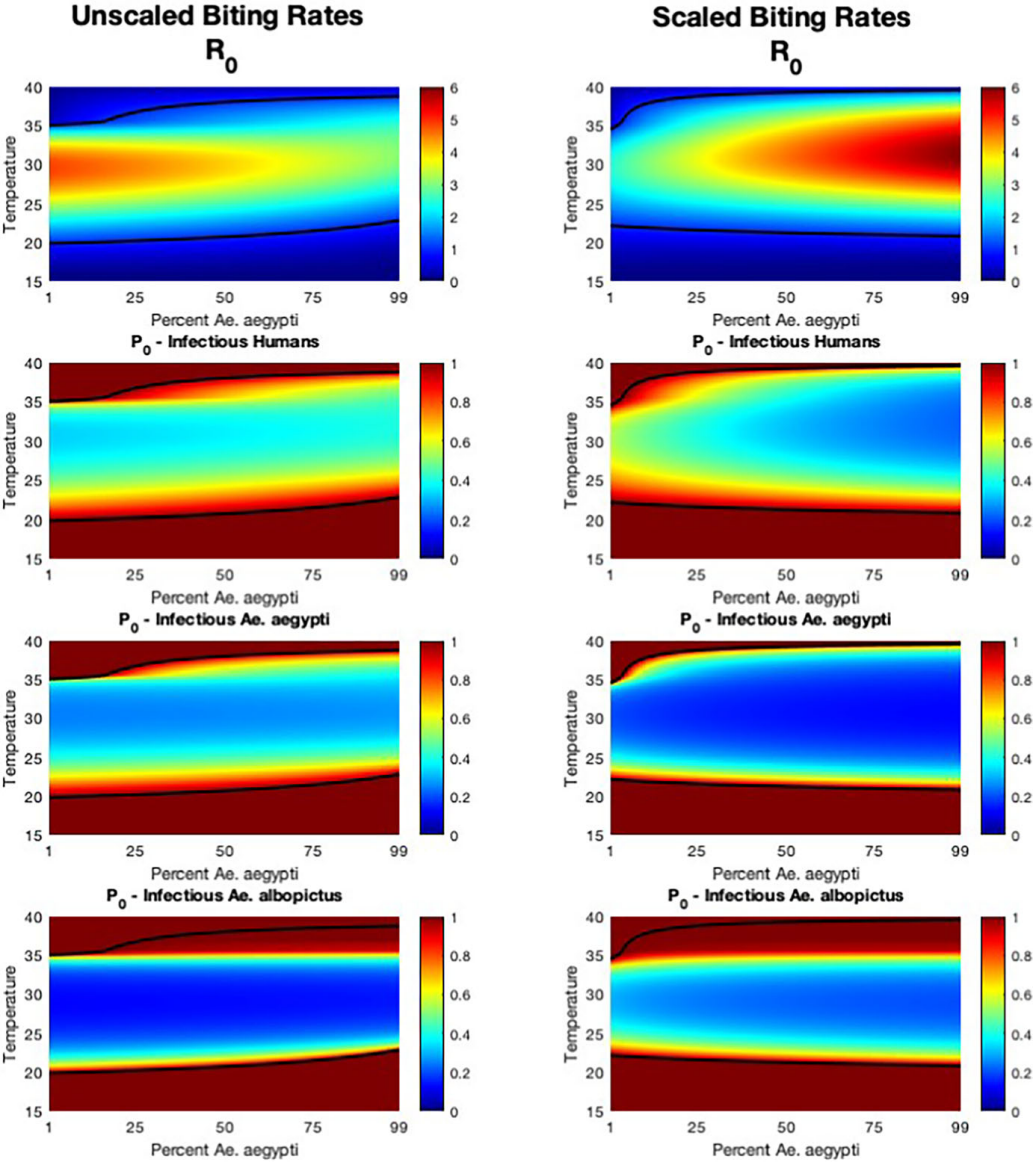
The effect of temperature and species composition on disease risk. The basic reproduction number $R_0$ and the probability of disease extinction $\mathbb {P}_0$ (for introduction by infectious humans, infectious *Ae. aegypti*, and infectious *Ae. albopictus*) are shown for temperatures from 15 to 40 C) and for 1% to 99% *Ae. aegypti*, and for two biting rate scenarios (unscaled and scaled). Black contours denote $R_0=1$ or $\mathbb {P}_0=1$. $N_{v_1} + N_{v_2} = 200$ and $N_h=100$ for a vector-host ratio of 2. All additional parameter values are described in Appendix A (Color figure online)

**Fig. 5:**
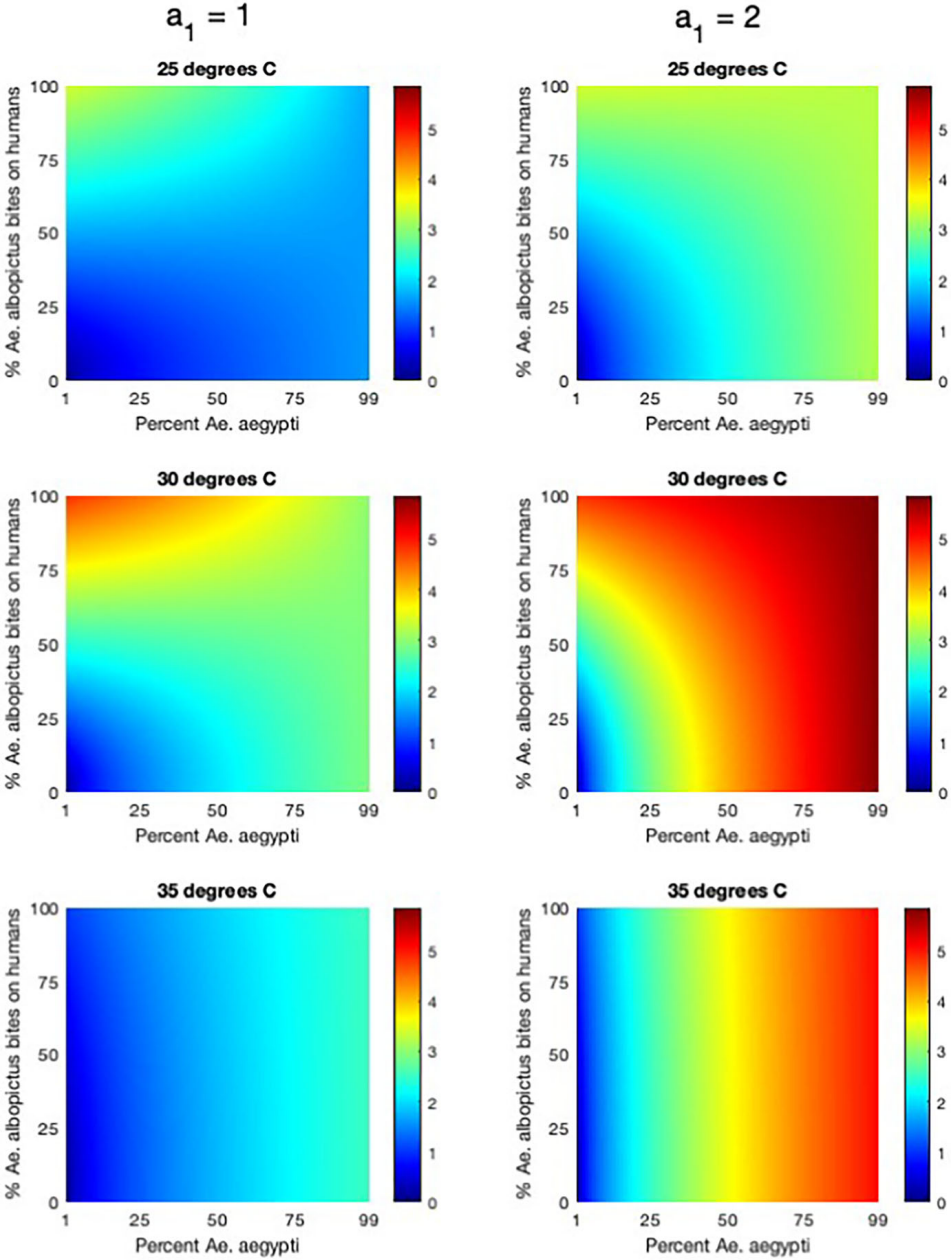
The effect of biting behavior on disease risk. $R_0$ is plotted as a function of vector composition (percent *Ae. aegypti* in the population) and the percentage of *Ae. albopictus* bites on humans. We show results for three temperatures (25, 30, and $35^{\circ }$C) for both unscaled ($a_1=1$) and scaled ($a_1=2$) assumptions for the biting rate of *Ae. aegypti*. We set $N_{v_1} + N_{v_2} = 200$ and $N_h=100$ for a vector-host ratio of 2. All additional parameter values are described in Appendix A (Color figure online)

At $35^{\circ }$C, the highest probability of extinction is for introduction by *Ae. albopictus*, for both unscaled and scaled biting rates. The lowest probability of extinction is for introduction by *Ae. aegypti* at low vector-host ratios, but once the vector-host ratio becomes large enough, the lowest probability of extinction is for introduction by humans. Across all temperatures, we again see that the probability of extinction for introduction by infected humans varies more with vector-host ratio than for introduction by any other type of infected individual.

### Temperature

Here we explore the effects of temperature on $R_0$ and $\mathbb {P}_0$. We consider a vector-host ratio of 2, composed of either 1%, 50%, or 99% *Ae. aegypti* (with the remainder of the vector population in each case being composed of *Ae. albopictus*), for both unscaled and scaled biting rates (Figure 3). When temperatures are too low or too high, $R_0$ falls below 1 and $\mathbb {P}_0$ is equal to 1 for introduction by all types of infectious individuals. The temperature for which $R_0$ first reaches 1 increases with the percentage of *Ae. aegypti* in the population for unscaled biting rates and decreases with the percentage of *Ae. aegypti* in the population for scaled biting rates. At higher temperatures, the temperature for which $R_0$ drops below 1 increases with the percentage of *Ae. aegypti*.

For intermediate temperatures, the lowest probability of extinction occurs for *Ae. albopictus* when biting rates are unscaled and for *Ae. aegypti* when biting rates are scaled. At higher temperatures the lowest probability of extinction occurs for introduction by *Ae. aegypti* for both scenarios. When *Ae. aegypti* only make up a small percentage of the population, the highest $\mathbb {P}_0$ values occur when the disease is introduced by humans. At higher temperatures (above $35^{\circ }$C), $\mathbb {P}_0$ decreases with the percentage of *Ae. aegypti* in the population, as long as the disease is not introduced by *Ae. albopictus*, as *Ae. albopictus* cannot survive long at that temperature.

### Temperature and Species Composition

In Figure 4, we further explore the combined impact of temperature and species composition on $R_0$ and $\mathbb {P}_0$. Horizontal cross-sections at temperatures of 25, 30, and $35^{\circ }$C recover Figure 1 while vertical cross sections at 1, 50, and 99% *Ae. aegypti* recover Figure 3. Contours where $R_0=\mathbb {P}_0=1$ are shown in black. To simplify presentation of our results, we focus only on disease introduction by infectious individuals rather than exposed.

When considering the impacts of both species composition and temperature, we find that $R_0$ values were highest with scaled biting rates and 100% of the mosquito population composed of *Ae. aegypti* (Figure 4). These values of $R_0$ occur between 30-$35^{\circ }$C. When biting rates are unscaled, $R_0$ values are highest when *Ae. albopictus* make up 100% of the population and temperatures are between 25-$31^{\circ }$C, although the highest $R_0$ values in these scenarios are still lower than those for scaled biting rates.

In considering $\mathbb {P}_0$ values, the lowest probability of extinction occurs either when *Ae. aegypti* introduces infection into a population composed mostly of *Ae. aegypti* and biting rates are scaled or when *Ae. albopictus* introduces infection into a population mostly composed of *Ae. albopictus* and biting rates are unscaled. For any given temperature, the percent composition does not have a substantial impact on the probabilities of extinction following introductions by mosquitoes; however, more variation is observed following introductions by humans. For example, at around $25^{\circ }$C, when biting rates are scaled, $\mathbb {P}_0$ is higher than 0.8 when the population is primarily composed of *Ae. albopictus*, but decreases to below 0.3 when the population is composed primarily of *Ae. aegypti*. We observe similar changes in the probability of extinction when temperatures are higher (around $35^{\circ }$C) in the unscaled biting rates scenario.

### Vector biting behavior

The actual percentage of *Ae. albopictus* bites on humans may vary depending on the availability of human hosts compared to alternative hosts independently of whether *Ae. aegypti* takes multiple bites per gonotrophic cycles. In Figure 5, we compare the effect of species composition (percent *Ae. aegypti*) and the percentage of *Ae. albopictus* bites on humans on $R_0$ for different assumptions about the biting rate of *Ae. aegypti* ($a_1$ either 1 (unscaled) or 2 (scaled) bites per gonotrophic cycle; see Appendix A.2) and for different temperatures (25, 30, or $35^{\circ }$C). When $a_1 = 1$, a horizontal cross section at 100% *Ae. albopictus* bites on humans corresponds to the previously explored unscaled biting rate scenario, and when $a_1 = 2$, a horizontal cross section at 50% *Ae. albopictus* bites on humans corresponds to the scaled biting rate scenario. Recall that for these scenarios, $R_0$ decreases with percent *Ae. aegypti* at 25 and $30^{\circ }$C with unscaled biting rates, but increases with percent *Ae. aegypti* at $35^\circ$C with unscaled biting rates and for 25, 30, and $30^{\circ }$C with scaled biting rates.

In Figure 5 we again see that $R_0$ increases with percent *Ae. aegypti* at $35^{\circ }$C with unscaled *Ae. aegypti* biting rates and for 25, 30, and $30^{\circ }$C with scaled *Ae. aegypti* biting rates regardless of the biting behavior of *Ae. albopictus*. However, when *Ae. aegypti* biting rates are unscaled, at the lower temperatures of 25 and $30^{\circ }$C we see $R_0$ decrease with percent *Ae. aegypti* if *Ae. albopictus* has a high enough biting rate on humans, but $R_0$ will increase with percent *Ae. aegypti* if the percentage of *Ae. albopictus* bites on humans is small.

At 30 C, when half of *Ae. albopictus* bites are on humans, we see very little effect on $R_0$ as we change the composition of the population if *Ae. aegypti* are assumed to have 1 bite per gonotrophic cycle. If we assume a larger *Ae. aegypti* biting rate of 2 bites per gonotrophic cycle, then we see a much larger effect on $R_0$ when we increase the percentage of *Ae. aegypti* in the population.

$R_0$ is always greater for $a_1 = 2$ compared to $a_1 = 1$, all else equal. For a given value of $a_1$ and set species composition, we generally see $R_0$ increase as the percent of *Ae. albopictus* bites on humans increases. The sensitivity of $R_0$ to the percentage of *Ae. albopictus* bites on humans decreases as the percent *Ae. aegypti* increases, meaning the percent of *Ae. albopictus* in the population decreases. The exception to this is that at higher temperatures ($35^{\circ }$C) where the lifespan of *Ae. albopictus* is short, we see little change in $R_0$ with the biting rate of *Ae. albopictus* regardless of the species composition of biting rate of *Ae. aegypti*.

## Discussion

In this paper, we developed a stochastic continuous time Markov chain model to extend our previous work exploring the effects of temperature and food quality on the outcome of competition between *Ae. aegypti* and *Ae. albopictus*, and the resulting risk of dengue using a deterministic model (Beck et al. 2025). In that work, we showed that at a given temperature, the abundance of each species present was seen to vary with food quality, habitat availability, and potentially initial conditions, and that these variables also impacted disease risk as measured by the basic reproduction number, $R_0$. Here, we further investigate the impacts of temperature, species composition, vector host ratio, biting behavior of both mosquito species, and source of initial introduction on the potential for disease transmission as measured by $R_0$ and the probability of disease extinction, $\mathbb {P}_0$.

Every set of parameter values considered gives a single estimate of $R_0$, based on disease introduction by a typical infected individual, and an estimate of $\mathbb {P}_0$ for introduction by each of the 6 types of infected individuals. We find that $R_0$ and $\mathbb {P}_0$ are generally inversely related for each type of infectious individual, with $P_0$ decreasing as $R_0$ increases or visa versa. However, the relative magnitudes of $\mathbb {P}_0$ for disease introduction by the different types of infected individuals vary greatly with model parameters, even when estimates of $R_0$ are similar. We show that overall, disease risk following introduction by humans is much more sensitive to vector composition than for introduction by vectors. This occurs because the model assumes an infectious vector will always find a host to bite, and the presence of other vectors of either species does not affect that process. However, if a human introduces the disease, then the vector composition—what vectors are present, what their biting rates are, and how many other potential humans to bite—are all important for determining whether that human will be bitten by a mosquito, and what the probability of passing the disease on to a vector would be.

These results have particular implications for interconnected regions with heterogeneity in temperature and mosquito species distributions. For example, in the city of New Orleans and surrounding suburbs, both *Ae. aegypti* and *Ae. albopictus* are present, but species abundance and composition varies by neighborhood (de Jesús Crespo R, Rogers RE 2021). This region, too, has many different microclimates, with a mixture of urban heat islands interspersed with cooler areas with more vegetation. Seasonal and long-term changes in temperature lead to shifts in species distributions, which, as we show here, could have important impacts for disease risk, with those impacts dependent on a number of other factors. For example, in a population with an average temperature of $25^{\circ }$C where *Ae. aegypti* and *Ae. albopictus* coexist in relatively similar ratios, a shift in the species composition that favors *Ae. albopictus* may lead to either larger risk of transmission and outbreaks (assuming unscaled biting rates) or a lower risk (assuming scaled biting rates) (Figure 4). If temperature also increases, this change in risk could be more pronounced.

It is likely that changes in temperature will also be accompanied by changes in species composition. For example, *Ae. albopictus* has higher fecundity and lower mortality rates than *Ae. aegypti* across most temperatures, but *Ae. aegypti* is more tolerant of higher temperatures, and previous work has shown that temperature-dependent risks associated with *Ae. aegypti* are higher than those of *Ae. albopictus* (Mordecai et al. 2017; Beck et al. 2025; Reinhold et al. 2018). With increased temperatures, areas currently dominated by *Ae. albopictus* could be replaced by *Ae. aegypti*, so that increases in temperature are accompanied by increases in the relative abundance of *Ae. aegypti*  which may lead to higher levels of transmission (scaled biting rates) or potentially lower rates of transmission (unscaled biting rates) (Laporta et al. 2023; Ryan et al. 2019).

While human movement is the primary driver of both short and long range mosquito-borne disease transmission (Stoddard et al. 2013; Horton and Robertson 2024), spread among interconnected neighborhoods in urban and/or metropolitan areas may be driven in part by movement of mosquitoes (Reiner et al. 2014; Chao et al. 2013). We show that the probability of outbreak is influenced by the species of the infected mosquito and that this probability is influenced by numerous factors including temperature and species composition. This could be of particular importance for informing mitigation strategies in urban areas where neighboring regions support different species. For example, if a cooler area with more vegetation borders a warmer, urban heat island, migration of infected *Ae. aegypti* mosquitoes from the urban heat island to the neighboring region could spark local outbreaks even if the *Ae. albopictus* population in that region cannot support sustained transmission of a pathogen.

Throughout this work, we emphasize the impact that vector biting behavior has on our results. In many cases, the assumption of whether biting rates are unscaled or scaled drastically changes disease risk. In Figure 5, we explore this further to show just how influential biting rate assumptions are on the results in combination with species composition and temperature. While *Ae. aegypti* may take multiple bites per gonotrophic cycle, the biting rate of the more opportunistic species *Ae. albopictus* on humans is more likely to vary spatially with the availability of human hosts compared to alternative hosts (i.e. in rural versus urban areas) (Niebylski et al. 1994; Fikrig et al. 2021). $R_0$ is particularly sensitive to the biting rate of *Ae. albopictus* at $30^{\circ }$C, where varying the percentage of bites that *Ae. albopictus* take on humans drives changes in $R_0$ from less than 1, with fewer bites on humans, to greater than 4, when most of their bites occurred on humans. The effect on $R_0$ is greatest when *Ae. albopictus* is the dominant species in the population, and even more pronounced when *Ae. aegypti* is assumed to take multiple bites per gonotrophic cycle. These results emphasize that better understanding of biting behaviors of *Ae. aegypti* and *Ae. albopictus* is paramount to studying the impacts of temperature and competition on mosquito-borne disease spread.

Our results have important implications for dengue control, particularly in regions where both *Ae. aegypti* and *Ae. albopictus* are implicated in transmission. We show that species composition influences $R_0$ as well as $\mathbb {P}_0$, with the specific effects on both dependent on biting rate assumptions (Figure 4). With the increasing promise of genetic and biological control strategies (Alphey et al. 2013; Benedict 2021; Oliva et al. 2021), species-specific control could result in changes to species composition in areas where both *Aedes* subspecies coexist. This could ultimately have the impact, for example, of reducing transmission at temperatures where *Ae. albopictus* are more dominant if they are replaced by *Ae. aegypti*  (Figure 4, unscaled biting rates), or increasing transmission if *Ae. aegypti* replace *Ae. albopictus* populations in other scenarios (Figure 4, scaled biting rates). Our work shows that careful consideration is necessary when considering species-targeted control measures in regions where both *Ae. aegypti* and *Ae. albopictus* are part of the dengue transmission cycle. Importantly, our results highlight that introductions of dengue by humans are most affected by species composition (Figure 4) and changes in vector-host ratio (Figure 2) when compared to introductions by either mosquito species. This result agrees with previous work suggesting that human movement plays a larger role in dengue spread than mosquito movement (Harish et al. 2024; Reiner et al. 2014; Stoddard et al. 2013), and suggests that human-focused control measures such as testing, quarantining, and vaccines may have the largest impact on reduction of transmission and the potential for outbreaks.

Our work makes some simplifying assumptions that should be further explored. Namely, we calculated $R_0$ and the probability of extinction $\mathbb {P}_0$ assuming a disease-free equilibrium at constant temperatures. A next step would be to incorporate seasonally varying or diurnal variations in temperature. We also acknowledge that some of the vector abundance and temperature scenarios we consider may be somewhat unrealistic, such as populations comprised of mostly *Ae. albopictus* at temperatures where the lifespan is very short and the species would likely not persist. However, since there are many factors affecting the outcome of competition between *Ae. aegypti* and *Ae. albopictus* that are not all well understood, this approach lets us focus on the implications of vector composition on disease risk, with computations for $R_0$ and $\mathbb {P}_0$ retaining the temperature dependence for vector life-history and disease-related parameters.

Taken together, our results emphasize the importance of temperature, species composition, vector-host ratio, mosquito biting behaviors, and source of initial introduction in the emergence and spread of dengue in a population where its two primary vectors share habitat. These results are critical in planning short- and long-term mitigation strategies in interconnected populations with high heterogeneity in human population, temperatures, and mosquito habitat availability. As we show here, small shifts in key variables may lead to important differences in species composition and disease risk, but our model and further investigation of this topic through similar models will be useful for better preparing for potential scenarios resulting from changes in these variables. Although this work has focused primarily dengue and *Aedes* subspecies, the framework presented here can be adapted to other mosquito-borne diseases where multiple mosquito species transmit pathogens and/or compete for resources.

## Data Availability

No datasets were generated or analyzed in this work. MATLAB code used to generate figures is available upon request.

## Parameters

Baseline model parameters are given below, see Beck et al. (2025) for additional details.

### Vector mortality rates

As in Beck et al. (2025), the mortality rates, measured in $\text {days}^{-1}$, for *Ae. aegypti* and *Ae. albopictus*, respectively, are taken to be the inverse of the average of curves fit to the $50 \%$ laboratory and field data for adult lifespan in Brady et al. (2013), with a minimum lifespan of 1 day assumed:

10 $$\begin{aligned} \mu _1(T) = \max {\left( \frac{2}{L_1^{\text {Lab}}(T) + L_1^{\text {Field}}(T)}, 1\right) } \end{aligned}$$

and

11 $$\begin{aligned} \mu _2(T) = \max {\left( \frac{2}{L_2^{\text {Lab}}(T) + L_2^{\text {Field}}(T)},1\right) } \end{aligned}$$

where

$$\begin{aligned} &  L_1^{\text {Field}}(T) = -0.007647T^2+0.3482T+1.844,\\ &  L_1^{\text {Lab}}(T) = -0.1194T^2+5.319T-18.16,\\ &  L_2^{\text {Field}}(T) =0.0005404T^4+0.04539T^3-1.41T^2+19.45T-89.68,\ \text {and}\\ &  L_2^{\text {Lab}}(T) = 0.0006021T^4-0.06474T^3+2.046T^2-19.26T+52. \end{aligned}$$

### Vector biting rates

As in Beck et al. (2025), we define the vector biting rates, measured in bites per mosquito per day, using Brière functions from Mordecai et al. (2017). We multiply the Brière functions by constant scaling parameters $a_1$ and $a_2$ to obtain the biting rates of *Ae. aegypti* and *Ae. albopictus* on humans, given by $b_1(T)$ and $b_2(T)$, respectively:

12 $$\begin{aligned} b_1(T) = a_1 0.000202T(T-13.35)(40.08-T)^{\frac{1}{2}} \end{aligned}$$

13 $$\begin{aligned} b_2(T) = a_2 0.000193T(T-10.25)(38.32-T)^{\frac{1}{2}} \end{aligned}$$

A value of $a_i$ less than 1 indicates that species *i* takes bites on non-human hosts, and a value of $a_i$ greater than 1 indicates that species *i* takes multiple bites per gonotrophic cycle. For the unscaled biting rate scenario, $a_1 = a_2 = 1$, and for the scaled biting rate scenario, $a_1 = 2$ and $a_2 = 0.5$.

### Host to vector transmission probability per bite

As in Beck et al. (2025), we define the per-bite probability of transmission from host to vector using Brière functions from Mordecai et al. (2017) with $\rho _{v_1}$ modified above $31.04^\circ$C to take on the value of the Brière function when $T=31.04$:

$$\begin{aligned} \rho _{v_1}(T)=&{\left\{ \begin{array}{ll} 0.000849T(T-17.05)(35.83-T)^{\frac{1}{2}}, & T < 31.04\\ 0.8069, & T \ge 31.04 \end{array}\right. }\\ \rho _{v_2}(T)=&\ 0.000735T(T-15.84)(36.4-T)^{\frac{1}{2}} \end{aligned}$$

### Vector to host transmission probability per bite

As in Beck et al. (2025), we define the per-bite probability of transmission from vector to host using Brière functions from Mordecai et al. (2017) with $\rho _{h_1}$ modified above $31.22^\circ$C to take on the value of the Brière function when $T=31.22$:

$$\begin{aligned} \rho _{h_1}(T) =&{\left\{ \begin{array}{ll} 0.000491T(T-12.22)(37.46-T)^{\frac{1}{2}}, & T < 31.22\\ 0.7275, & T \ge 31.22. \end{array}\right. }\\ \rho _{h_2}(T) =&\ 0.000439T(T-3.62)(36.82-T)^{\frac{1}{2}} \end{aligned}$$

### Vector extrinsic incubation rate

As in Beck et al. (2025), we define the extrinsic incubation rate, measured in $\text {days}^{-1}$, for dengue using Brière functions from Mordecai et al. (2017):

$$\begin{aligned} \sigma _1(T)&= 0.0000665T(T-10.68)(45.9-T)^{\frac{1}{2}}\\ \sigma _2(T)&= 0.000109T(T-10.39)(43.05-T)^{\frac{1}{2}} \end{aligned}$$

### Host parameters

As in Beck et al. (2025), the host parameters are assumed to be temperature independent with values taken from Huber et al. (2018).

Host intrinsic incubation period: $\sigma _h = 1/5.9$ ($\text {days}^{-1}$)

Human recovery rate: $r_h = 1/5$ ($\text {days}^{-1}$)

Birth/Death rate of humans: $\mu _h = 1/(70*365)$ ($\text {days}^{-1}$)

## Relating the Deterministic and Stochastic Thresholds

From the deterministic model (1), we can define the vector of infectious classes $X = (E_h, I_h, E_{v_1}, I_{v_1}, E_{v_2}, I_{v_2})$. The linearization at the disease free equilibrium is

$$\begin{aligned} \frac{dX}{dt}=(F-V)X \end{aligned}$$

where

14 $$\begin{aligned} F= \begin{bmatrix} 0 & 0 & 0 & b_1(T) \rho _{h_1}(T)& 0 & b_2(T) \rho _{h_2}(T) \\ 0 & 0 & 0 & 0 & 0 & 0\\ 0 & \frac{b_1(T) \rho _{v_1}(T) N_{v_1}}{N_h} & 0 & 0& 0 & 0\\ 0 & 0 & 0 & 0 & 0 & 0\\ 0 & \frac{b_2(T) \rho _{v_2}(T) N_{v_2}}{N_h} & 0 & 0& 0 & 0\\ 0 & 0 & 0 & 0 & 0 & 0\\ \end{bmatrix} \end{aligned}$$

and

15 $$\begin{aligned} V = \begin{bmatrix} \sigma _h + \mu _h & 0 & 0 & 0 & 0 & 0\\ -\sigma _h & r_h + \mu _h & 0 & 0 & 0 & 0 \\ 0 & 0 & \sigma _1(T) + \mu _1(T) & 0 & 0 & 0\\ 0 & 0& -\sigma _1(T) & \mu _1(T) & 0 & 0 \\ 0 & 0 & 0 & 0 & \sigma _2(T) + \mu _2(T) & 0\\ 0 & 0 & 0 & 0 & -\sigma _2(T) & \mu _2(T) \\ \end{bmatrix}. \end{aligned}$$

The basic reproduction number $R_0$ of the deterministic model is the spectral radius of the second generation matrix $FV^{-1}$.

From the offspring PGFs for the 6 types of infectious individuals (4)-(9), we can define a 6-by-6 expectation matrix, $\mathbb {M}$, where the (*j*, *i*) entry $\displaystyle \frac{\partial {f_i}}{\partial {u_j}}$ evaluated at the fixed point $(u_1,u_2,u_3,u_4,u_5,u_6) = (1,1,1,1,1,1)$ gives the expected number of new infections of type *j* produced by an infected individual of type *i* (Allen and Lahodny 2012):

16 $$\begin{aligned} \mathbb {M} = \begin{bmatrix} 0 & 0 & 0 & \dfrac{b_1(T) \rho _{h_1}(T)}{b_1(T) \rho _{h_1}(T) + \mu _1(T)} & 0 & \dfrac{b_2(T) \rho _{h_2}(T)}{b_2 \rho _{h_2}(T) + \mu _2(T)} \\ \dfrac{\sigma _h}{\sigma _h+\mu _h} & M_{22} & 0 & 0 & 0 & 0 \\ 0 & M_{32} & 0 & 0 & 0 & 0 \\ 0 & 0 & \dfrac{\sigma _1(T)}{\sigma _1(T) + \mu _1(T)} & \dfrac{b_1(T) \rho _{h_1}(T)}{b_1(T) \rho _{h_1}(T) + \mu _1(T)} & 0 & 0 \\ 0 & M_{52} & 0 & 0 & 0 & 0 \\ 0 & 0 & 0 & 0 & \dfrac{\sigma _2(T)}{\sigma _2(T) + \mu _2(T)} & \dfrac{b_2(T) \rho _{h_2}(T)}{b_2 \rho _{h_2}(T) + \mu _2(T)} \\ \end{bmatrix}. \end{aligned}$$

where

$$\begin{aligned} M_{22} = \dfrac{\frac{b_1(T) \rho _{v_1}(T) N_{v_1}}{N_h} + \frac{b_2(T) \rho _{v_2}(T) N_{v_2}}{N_h}}{\frac{b_1(T) \rho _{v_1}(T) N_{v_1}}{N_h} + \frac{b_2(T) \rho _{v_2}(T) N_{v_2}}{N_h} + r_h + \mu _h}, \end{aligned}$$

$$M_{32} = \dfrac{\frac{b_1(T) \rho _{v_1}(T) N_{v_1}}{N_h}}{\frac{b_1(T) \rho _{v_1}(T) N_{v_1}}{N_h} + \frac{b_2(T) \rho _{v_2}(T) N_{v_2}}{N_h} + r_h + \mu _h},$$

and

$$M_{52} = \dfrac{\frac{b_2(T) \rho _{v_2}(T) N_{v_2}}{N_h}}{\frac{b_1(T) \rho _{v_1}(T) N_{v_1}}{N_h} + \frac{b_2(T) \rho _{v_2}(T) N_{v_2}}{N_h} + r_h + \mu _h}.$$

$\mathbb {M}$ is non-negative and irreducible, and satisfies the relationship $(\mathbb {M}-\mathbb {I})W = F-V$, where $\mathbb {I}$ is the $6 \times 6$ identity matrix, $W = {{\,\textrm{diag}\,}}(\sigma _h+\mu _h,\frac{b_1(T) \rho _{v_1}(T) N_{v_1}}{N_h} + \frac{b_2(T) \rho _{v_2}(T) N_{v_2}}{N_h} + r_h + \mu _h,\sigma _1(T)+\mu _1(T),b_1(T)\rho _{h_1}(t)+\mu _1(T),\sigma _2(T)+\mu _2(T),b_2(T)\rho _{h_2}(t)+\mu _2(T))$, and *F* and *V* are the matrices above in equations (14) and (15). Therefore the conditions for the Threshold Theorem from Allen and van den Driessche (2013) to apply are met, guaranteeing $R_0 < 1 (=1, >1)$ if and only if $\rho (\mathbb {M}) < 1 (=1, >1)$, as well as the existence of a unique fixed point of the offspring PGFs (4)-(9), $(q_1,q_2,q_3,q_4,q_5,q_6) \in (0,1)^6$ when the branching process is supercritical.

## References

[CR1] Allen LJ (2010) An Introduction to Stochastic Processes with Applications to Biology. CRC Press

[CR2] Allen LJ (2017) A primer on stochastic epidemic models: Formulation, numerical simulation, and analysis. Infect Dis Model 2(2):128–142 10.1016/j.idm.2017.03.001 PMC6002090 29928733

[CR3] Allen LJ, van den Driessche P (2013) Relations between deterministic and stochastic thresholds for disease extinction in continuous-and discrete-time infectious disease models. Math Biosci 243(1):99–108 23458509 10.1016/j.mbs.2013.02.006

[CR4] Allen LJ, Lahodny GE Jr (2012) Extinction thresholds in deterministic and stochastic epidemic models. J Biol Dyn 6(2):590–611 22873607 10.1080/17513758.2012.665502

[CR5] Alphey L, McKemey A, Nimmo D, Neira Oviedo M, Lacroix R, Matzen K, Beech C (2013) Genetic control of *Aedes* mosquitoes. Pathog Glob Health 107(4):170–179 10.1179/2047773213Y.0000000095 PMC4001467 23816508

[CR6] Beck E, Beuerle L, Martin P, Stambaugh R, de Jesús Crespo R, Robert MA, Robertson SL (2025) Modeling the effects of temperature and resource quality on the outcome of competition between *Aedes aegypti* and *Aedes albopictus* and the resulting risk of vector-borne disease: Bullet Math Biol **87**(10), 138 10.1007/s11538-025-01518-x PMC12398451 40884588

[CR7] Benedict MQ (2021) Sterile insect technique: lessons from the past. J Med Entomol 58(5):1974–1979 33629719 10.1093/jme/tjab024

[CR8] Brady OJ, Johansson MA, Guerra CA, Bhatt S, Golding N, Pigott DM, Delatte H, Grech MG, Leisnham PT, Maciel-de-Freitas R et al (2013) Modelling adult *Aedes aegypti* and *Aedes albopictus* survival at different temperatures in laboratory and field settings. Parasit Vectors 6:1–12 10.1186/1756-3305-6-351 PMC3867219 24330720

[CR9] Camara DCP, Codeço CT, Juliano SA, Lounibos LP, Riback TIS, Pereira GR, Honorio NA (2016) Seasonal differences in density but similar competitive impact of *Aedes albopictus* (skuse) on *Aedes aegypti* (l.) in Rio de Janeiro, Brazil. PLoS One **11**(6), 0157120 10.1371/journal.pone.0157120 PMC4913923 27322537

[CR10] Chao DL, Longini IM Jr, Halloran ME (2013) The effects of vector movement and distribution in a mathematical model of dengue transmission. PLoS ONE 8(10):76044 10.1371/journal.pone.0076044 PMC3804532 24204590

[CR11] de Jesús Crespo R, Rogers RE (2021) Habitat segregation patterns of container breeding mosquitos: The role of urban heat islands, vegetation cover, and income disparity in cemeteries of New Orleans. Int J Environ Res Public Health 19(1):245 10.3390/ijerph19010245 PMC8751023 35010505

[CR12] Ding F, Fu J, Jiang D, Hao M, Lin G (2018) Mapping the spatial distribution of *Aedes aegypti* and *Aedes albopictus*. Acta Trop 178:155–162 10.1016/j.actatropica.2017.11.020 29191515

[CR13] Fader JE (2016) The importance of interspecific interactions on the present range of the invasive mosquito *Aedes albopictus* (Diptera: Culicidae) and persistence of resident container species in the United States. J Med Entomol 53(5):992–1001 10.1093/jme/tjw095 27354436

[CR14] Fikrig K, Martin E, Dang S, St Fleur K, Goldsmith H, Qu S, Rosenthal H, Pitcher S, Harrington LC (2021) The effects of host availability and fitness on *Aedes albopictus* blood feeding patterns in new york. Am J Trop Med Hyg 106(1):320 10.4269/ajtmh.21-0157 PMC8733534 34662859

[CR15] Harish V, Colón-González FJ, Moreira FR, Gibb R, Kraemer MU, Davis M, Reiner RC Jr, Pigott DM, Perkins TA, Weiss DJ et al (2024) Human movement and environmental barriers shape the emergence of dengue. Nat Commun 15(1):4205 38806460 10.1038/s41467-024-48465-0 PMC11133396

[CR16] Hawley WA, Reiter P, Copeland RS, Pumpuni CB, Craig GB Jr (1987) *Aedes albopictus* in North America: probable introduction in used tires from northern Asia. Science 236(4805):1114–1116 10.1126/science.3576225 3576225

[CR17] Honório NA, Silva WdC, Leite PJ, Gonçalves JM, Lounibos LP, Lourenço-de-Oliveira R (2003) Dispersal of *Aedes aegypti* and *Aedes albopictus* (Diptera: Culicidae) in an urban endemic dengue area in the state of Rio de Janeiro, Brazil. Memórias do Instituto Oswaldo Cruz **98**, 191–198 10.1590/s0074-02762003000200005 12764433

[CR18] Horton EB, Robertson SL (2024) A stochastic multi-host model for West Nile virus transmission. J Biol Dyn 18(1):2293780 38153263 10.1080/17513758.2023.2293780

[CR19] Huber JH, Childs ML, Caldwell JM, Mordecai EA (2018) Seasonal temperature variation influences climate suitability for dengue, chikungunya, and zika transmission. PLoS Negl Trop Dis 12(5):0006451 10.1371/journal.pntd.0006451 PMC5963813 29746468

[CR20] Juliano SA, Lounibos LP, O’Meara GF (2004) A field test for competitive effects of *Aedes albopictus* on *A. aegypti* in South Florida: differences between sites of coexistence and exclusion? Oecologia **139**, 583–593 10.1007/s00442-004-1532-4 PMC1906877 15024640

[CR21] Juliano SA (2010) Coexistence, exclusion, or neutrality? a meta-analysis of competition between *Aedes albopictus* and resident mosquitoes. Isr J Ecol Evol 56(3–4):325–351 10.1560/IJEE.55.3-4.325 PMC3589801 23482823

[CR22] Kraemer MU, Reiner RC Jr, Brady OJ, Messina JP, Gilbert M, Pigott DM, Yi D, Johnson K, Earl L, Marczak LB et al (2019) Past and future spread of the arbovirus vectors *Aedes aegypti* and *Aedes albopictus*. Nat Microbiol 4(5):854–863 10.1038/s41564-019-0376-y PMC6522366 30833735

[CR23] Lahodny GE Jr, Allen LJ (2013) Probability of a disease outbreak in stochastic multipatch epidemic models. Bull Math Biol 75:1157–1180 23666483 10.1007/s11538-013-9848-z

[CR24] Lambrechts L, Scott TW, Gubler DJ (2010) Consequences of the expanding global distribution of *Aedes albopictus* for dengue virus transmission. PLoS Negl Trop Dis 4(5):646 10.1371/journal.pntd.0000646 PMC2876112 20520794

[CR25] Laporta GZ, Potter AM, Oliveira JF, Bourke BP, Pecor DB, Linton Y-M (2023) Global distribution of *Aedes aegypti* and *Aedes albopictus* in a climate change scenario of regional rivalry. Insects 14(1):49 10.3390/insects14010049 PMC9860750 36661976

[CR26] Lloyd AL, Zhang J, Root AM (2007) Stochasticity and heterogeneity in host-vector models. J R Soc Interface 4(16):851–863 17580290 10.1098/rsif.2007.1064 PMC2394551

[CR27] Lounibos L (2007) Competitive displacement and reduction. J Am Mosq Control Assoc 23(2 Suppl):276 10.2987/8756-971x(2007)23[276:cdar]2.0.co;2 PMC2212597 17853612

[CR28] Lounibos LP, Juliano SA (2018) Where vectors collide: the importance of mechanisms shaping the realized niche for modeling ranges of invasive *Aedes* mosquitoes. Biol Invasions 20(8):1913–1929 10.1007/s10530-018-1674-7 PMC6133263 30220875

[CR29] Lozano-Fuentes S, Kenney JL, Varnado W, Byrd BD, Burkhalter KL, Savage HM (2019) Susceptibility and vectorial capacity of american *Aedes albopictus* and *Aedes aegypti* (diptera: Culicidae) to american zika virus strains. J Med Entomol 56(1):233–240 10.1093/jme/tjy114 PMC6781865 30102327

[CR30] Maliyoni M (2020) Probability of disease extinction or outbreak in a stochastic epidemic model for West Nile virus dynamics in birds. Acta Biotheoretica, 1–26 10.1007/s10441-020-09391-y 32889647

[CR31] Manore CA, Hickmann KS, Xu S, Wearing HJ, Hyman JM (2014) Comparing dengue and chikungunya emergence and endemic transmission in *A. aegypti* and *A. albopictus*. J Theor Biol**356**, 174–191 10.1016/j.jtbi.2014.04.033 PMC4109365 24801860

[CR32] Moore CG et al (1999) *Aedes albopictus* in the United States: current status and prospects for further spread. J Am Mosq Control Assoc 15(2):221–227 10412117

[CR33] Mordecai EA, Cohen JM, Evans MV, Gudapati P, Johnson LR, Lippi CA, Miazgowicz K, Murdock CC, Rohr JR, Ryan SJ et al (2017) Detecting the impact of temperature on transmission of zika, dengue, and chikungunya using mechanistic models. PLoS Negl Trop Dis 11(4):0005568 10.1371/journal.pntd.0005568 PMC5423694 28448507

[CR34] Niebylski ML, Savage HM, Nasci RS, Craig GB Jr (1994) Blood hosts of *Aedes albopictus* in the United States. J Am Mosq Control Assoc 10(3):447–450 7807094

[CR35] O’Dell N, Bolling BG, Dacko N, Carr JT, Hambrick B, Chaves LF, McMillan JR (2025) Identifying environmental drivers of *Aedes aegypti* and *Aedes albopictus* abundance in the Dallas-Fort Worth metroplex using random forest modeling. J Med Entomol, 036 10.1093/jme/tjaf036 PMC12271727 40209100

[CR36] Oliva CF, Benedict MQ, Collins CM, Baldet T, Bellini R, Bossin H, Bouyer J, Corbel V, Facchinelli L, Fouque F et al (2021) Sterile insect technique (SIT) against *Aedes* species mosquitoes: A roadmap and good practice framework for designing, implementing and evaluating pilot field trials. Insects 12(3):191 10.3390/insects12030191 PMC7996155 33668374

[CR37] O’Meara GF, Evans Jr LF, Gettman AD, Cuda JP (1995) Spread of *Aedes albopictus* and decline of *Ae. aegypti* (Diptera: Culicidae) in Florida. J Med Entomol **32**(4), 554–562 10.1093/jmedent/32.4.554 7650719

[CR38] Reiner RC Jr, Stoddard ST, Scott TW (2014) Socially structured human movement shapes dengue transmission despite the diffusive effect of mosquito dispersal. Epidemics 6:30–36 24593919 10.1016/j.epidem.2013.12.003 PMC3971836

[CR39] Reinhold JM, Lazzari CR, Lahondère C (2018) Effects of the environmental temperature on *Aedes aegypti* and *Aedes albopictus* mosquitoes: a review. Insects 9(4):158 10.3390/insects9040158 PMC6316560 30404142

[CR40] Rezza G (2012) *Aedes albopictus* and the reemergence of dengue. BMC Public Health 12:1–3 10.1186/1471-2458-12-72 PMC3398301 22272602

[CR41] Robert MA, Christofferson RC, Weber PD, Wearing HJ (2019) Temperature impacts on dengue emergence in the united states: Investigating the role of seasonality and climate change. Epidemics 28:100344 31175008 10.1016/j.epidem.2019.05.003 PMC6791375

[CR42] Ryan SJ, Carlson CJ, Mordecai EA, Johnson LR (2019) Global expansion and redistribution of *Aedes*-borne virus transmission risk with climate change. PLoS Negl Trop Dis 13(3):0007213 10.1371/journal.pntd.0007213 PMC6438455 30921321

[CR43] Sangermano F, Asar ML, Visintin AM, Benítez E, Ludueña-Almeida FF, Estallo EL (2025) Temporal and spatial dynamics of urban heat in the city of Córdoba, Argentina. J Clim Chang Health 26:100599 10.1016/j.joclim.2025.100599 PMC12851193 41647848

[CR44] Scott TW, Clark GG, Lorenz LH, Amerasinghe PH, Reiter P, Edman JD (1993) Detection of multiple blood feeding in *Aedes aegypti* (Diptera: Culicidae) during a single gonotrophic cycle using a histologic technique. J Med Entomol 30(1):94–99 10.1093/jmedent/30.1.94 8433350

[CR45] Sivan A, Shriram A, Sunish I, Vidhya P (2015) Host-feeding pattern of *Aedes aegypti* and *Aedes albopictus* (diptera: Culicidae) in heterogeneous landscapes of South Andaman, Andaman and Nicobar Islands, India. Parasitol Res 114:3539–3546 10.1007/s00436-015-4634-5 26220560

[CR46] Stoddard ST, Forshey BM, Morrison AC, Paz-Soldan VA, Vazquez-Prokopec GM, Astete H, Reiner RC Jr, Vilcarromero S, Elder JP, Halsey ES et al (2013) House-to-house human movement drives dengue virus transmission. Proc Natl Acad Sci 110(3):994–999 23277539 10.1073/pnas.1213349110 PMC3549073

[CR47] Vega-Rúa A, Zouache K, Girod R, Failloux A-B, Lourenço-de-Oliveira R (2014) High level of vector competence of *Aedes aegypti* and *Aedes albopictus* from ten american countries as a crucial factor in the spread of chikungunya virus. J Virol 88(11):6294–6306 10.1128/JVI.00370-14 PMC4093877 24672026

[CR48] Whitehorn J, Kien DTH, Nguyen NM, Nguyen HL, Kyrylos PP, Carrington LB, Tran CNB, Quyen NTH, Thi LV, Le Thi D et al (2015) Comparative susceptibility of *Aedes albopictus* and *Aedes aegypti* to dengue virus infection after feeding on blood of viremic humans: implications for public health. J Infect Dis 212(8):1182–1190 10.1093/infdis/jiv173 PMC4577038 25784733

[CR49] Yee DA, Kesavaraju B, Juliano SA (2004) Interspecific differences in feeding behavior and survival under food-limited conditions for larval *Aedes albopictus* and *Aedes aegypti* (diptera: Culicidae). Ann Entomol Soc Am 97(4):720–728 10.1603/0013-8746(2004)097[0720:IDIFBA]2.0.CO;2 PMC3507448 23197877

